# The Greek versions of the TeamSTEPPS teamwork perceptions questionnaire and Minnesota satisfaction questionnaire “short form”

**DOI:** 10.1186/s12913-020-05451-8

**Published:** 2020-06-26

**Authors:** Ioanna Lakatamitou, Ekaterini Lambrinou, Martha Kyriakou, Lefkios Paikousis, Nicos Middleton

**Affiliations:** Department of Nursing, Scholl of Health Sciences, Cyprus University of Technology, 15, Vragadinou str, 3041 Limassol, Cyprus

**Keywords:** Teamwork, Job satisfaction, Validation, Tools, Gr-T-TPQ, Gr-MSQ-short

## Abstract

**Background:**

Teamwork and job satisfaction are important among the multidisciplinary team who care patients with chronic illnesses such as heart failure (HF) patients. TeamSTEPPS teamwork perceptions questionnaire (T-TPQ) and Minnesota Satisfaction Questionnaire “short form” (MSQ-short) are both self-report questionnaires which examine multiple dimensions of perceptions of teamwork and job satisfaction within healthcare settings, respectively. The aim of the study was to examine the psychometric properties of the Greek versions of the TeamSTEPPS Teamwork perceptions questionnaire (Gr-T-TPQ) and Minnesota Satisfaction Questionnaire “short form” (Gr-MSQ-short).

**Methods:**

A methodological study was contacted in order to assess the construct validity and reliability of the Gr-T-TPQ and Gr-MSQ-short. For that reason, 292 questionnaires were administrated to Greek-Cypriot health care professionals (HCPs). Confirmatory factor analysis (CFA) was conducted for the data collected with the GrT-TPQ and Exploratory factor analysis (EFA) and CFA were also conducted for the data collected with the GrMSQ-short questionnaire. Cronbach’s a was calculated as well.

**Results:**

CFA of the data collected with Gr-T-TPQ confirmed the initial scale structure with excellent fit indices (× 2 (df) 1124.75 (550), p < 0.0001, AGFI = 0.986, TLI = 0.994, CFI = 0.994, RMSEA = 0.06, 90%, C.I.[0.055–0.065]). Furthermore, all dimensions were found to be correlated (r = 0.65 to r = 0.88) and internal consistency was found adequate (Cronbach’s α = 0.96). Subscales also, demonstrated high internal consistency (α = 0.87–0.95). CFA for the data collected with Gr-MSQ-short, did not confirm the initial scale’s dimensions. In EFA items 1, 5, 6, 12 and 18 were eliminated from the analysis due to low communalities and multiple components loading. The oblimin rotation with two factors was explaining 58% of the variance. These two factors identified were Supervisor/Autonomy and Task Enrichment.

**Conclusions:**

In general, the Gr-T-TPQ and Gr-MSQ-short are construct-valid instruments for measuring perceptions of teamwork and job satisfaction in Greek speaking HCPs’ population.

## Background

The strong association between the well-being of health care professionals (HCPs) and patients’ outcomes has been previously proved [1–3]. Burnout is described as a psychological syndrome, resulting from exposure to chronic emotional and interpersonal workplace stressors [4]. HCPs are often exposed to different psychosocial hazards that originate from the workplace demands and conditions. Moreover, there are different physical, social, emotional, cognitive, and organizational factors that require prolonged physical or psychological efforts from HCPs. On the other hand, job resources such as good salary, teamwork, job satisfaction, participation in decision making, performance feedback and team building activities may help HCPs in achieving work goals [5–7].

Job satisfaction of all HCPs in health care settings has been broadly investigated. It is an emotional condition of individuals, strengthened by positive feelings and feelings of belonging to a workplace mode in a particular workplace [2]. Reduced level of job satisfaction is a major cause of reversals at work, particularly in the area of health care services [8, 9]. Job satisfaction is found to affect quality of care and health care services. Studies have shown that, job satisfaction is a major predictor of the absences of HCPs, burnout and intention of resigning from their jobs. Moreover, HCP’s job satisfaction, indirectly affect patients’ satisfaction, quality of care and mortality. It may also affect the organizational commitment [10, 11].

Furthermore, teamwork is found to be essential for a safer and more effective health care system [12–14]. The discourse of teamwork has become a defining feature of healthcare reform. Policy makers, HCPs and researchers have increasingly emphasized teamwork as the way through which safe and patient-centered outcomes can be achieved [12, 13]. Being part of a team or working as a team is not automatically successful [8]. Estimation of HCPs teamwork and job satisfaction perceptions require the use of valid and reliable questionnaires before attempting any interventions on improving these aspects of job [12, 13].

Literature search revealed few questionnaires measuring teamwork attitudes, oriented to health care settings. These are the Cockpit Management Attitudes Questionnaire [15], the Safety Attitudes Questionnaire measures attitudes [16] and TeamSTEPPS teamwork perceptions questionnaire (T-TPQ) [17]. T-TPQ is a self-reported tool which measures teamwork within a unit or a department. The certain tool was chosen for the current study because it is a comprehensive tool and includes all components of teamwork: team structure, leadership, communication, mutual support, and situation monitoring and are relevant to teams working in dynamic and complex environments, such as hospitals. Moreover, it is found to be relative to environments where impacts of mistakes are high and the chance for misunderstanding is low [17]. It is quite a new tool and has only been validated in USA populations showing adequate reliability and validity components when assessing teamwork perceptions in health care settings [17, 18].

Numerous scales have been developed assessing job satisfaction on both; HCPs and other professions as well. In this study, Minnesota Satisfaction Questionnaire - Short form (MSQ-short) was chosen [19]. MSQ-short is a well-known instrument over the time [19–22]. MSQ-short is also a self-completed tool which measures employees’ job satisfaction. It is a multidimensional tool, allowing a more comprehensive understanding of the issue assessed. Previous validations have shown very good alfa coefficient values [20–23].

## Methods

### Aim of the study

The aim of the study is to evaluate the psychometric properties of the Greek version of the T-TPQ (Gr-T-TPQ) and MSQ-short (Gr-MSQ-short) in a Greek-Cypriot population, by assessing the construct validity, the internal consistency and factor reliability of the scales and factors.

### Design and ethical considerations

The methodological study was designed as a cross sectional survey. Approvals were shaught and granted by the Cyprus Bioethics Committee and all parties involved. The administration of each hospital reviewed the study protocol and agreed to the implementation of the study. All procedures were in line with the instructions given by the Data Protection Commissioner for maintaining confidentiality. The study involved no risk or harm to the subjects and the participation of the sample was voluntarily.

### Setting and sample

The sample consisted of Greek-Cypriot HCPs. HCPs were recruited from random units and departments of the four large public hospitals and two private hospitals of Cyprus. The main researcher approached HCPs in their workplaces and restricted the participation to those who provide written consent. HCPs who did not speak Greek were excluded. Authors wanted to assess translation and adaptation of the translation, so it was important the sample could understand the meaning of the words and whether it made sense. Questionnaires were administered by the researcher who supervised the completion procedure. Several socio-demographic variables e.g. gender, age, professional and educational status, were also provided through a short self-completed questionnaire. In order to ensure at least 8:1 participant to items ratio, the sample size was set to a minimum of 280 participants.

### The TeamSTEPPS-teamwork perceptions questionnaire

T-TPQ is a 5-point Likert scale, from 1 (“Strongly agree”) to 5 (“Strongly disagree”), that measures HCPs teamwork perceptions. The total score is calculated by summing the ratings for each item (35 items) and ranges from 35 to 175, with higher scores indicating poorer teamwork perceptions. It includes five subscales with seven items each, which can be used separately. Team Structure, Leadership, Situation Monitoring, Mutual Support and Communication [17].

### The Minnesota satisfaction questionnaire “short form”

MSQ-short is a 5-point Likert scale, from 1 (“Very dissatisfied with this aspect of my job”) to 5 (“Very satisfied with this aspect of my job”), that measures HCPs’ job satisfaction perceptions. It is created by a bigger scale which contained 100 questions (20 categories), by taking the question which best represent each of the categories. The total score is calculated by summing the ratings for each item (20 items). The total score ranges from 20 to 100, with higher scores indicating higher job satisfaction perceptions. It includes two subscales, intrinsic and extrinsic satisfaction. It was chosen by the researchers, although an old tool, as it provides more specific and in detail information on the aspects of a job that an individual finds rewarding. Also, it is an easy self-reported questionnaire which takes little time to be completed (approximately five minutes) [19].

### Translation and equivalence of the Greek versions

Permission to translate and use the English versions of the T-TPQ and MSQ-short was obtained from the authors of the original questionnaires. The process followed the classic approach of translation and back-translation (Brislin’s model 1970). Two bilingual nurses translated the questionnaires from English into the Greek language while two other bilingual nurses undertook the back-translation. A research team consisting of five bilingual nurses reviewed the differences of the back-translation in order to establish semantic equivalence. All the members of the team agreed to the final versions. For assessing the readability of the final versions, three HCPs were asked to appraise the Greek versions. No difficulties were encountered in understanding or in explaining the items of the Gr-T-TPQ and Gr-MSQ-short.

### Statistical analysis

Descriptive statistics were used to describe the demographic characteristics of the participants. Content validity was indirectly assessed by a panel of experts who evaluated the suitability of the Greek translation. Construct validity was assessed by a factor analysis of the data collected. Confirmatory factor analysis (CFA) was performed in order to evaluate the fit on the assumed theoretical dimensions of the Gr-T-TPQ and Gr-MSQ-short, as proposed by Baker et al. (2010) [14] and Weiss et al. (1967) [6], respectively. In the CFA analysis, modification indices were used for model improvement. In the case that CFA would not suggest an adequate data model fit, an exploratory factor analysis (EFA) was decided to be performed. A range of goodness of fit criteria to assess the overall model fit were used including root mean square error of approximation (RMSEA) < 0.08, goodness of fit index (GFI) > 0.90, adjusted goodness of fit index (AGFI) > 0.90, normed fit index (NFI) > 0.90 and comparative fit index (CFI) > 0.90. Kaiser Meyer Olkin coefficient and Bartlett’s test of sphericity were used to verify the adequacy of the data for factor analysis. EFA with oblique rotation and Kaiser normalization were applied to extract the factors. The cut-off point for the factor loadings was set at 0.40, in order to reduce cross-loading of items on several factors. In terms of reliability, the internal consistency of the Gr-T-TPQ and the Gr-MSQ-short as whole scales and across the identified factors was investigated by calculating composite reliability [24] and Cronbach’s alpha coefficients. Both tools were re-administered to a random sample of 15 participants after a period of 2 weeks from the initial assessment. Pearson correlation analysis was conducted to validate the stability of the instruments (test-retest reliability). Analysis was performed in the open source statistical software *R* [25] and the package *lavaan* [26].

## Results

### Description of sample

Questionnaires were given and collected completed to 292 nurses with response rate 100%. The demographic and clinical characteristics of the 292 participants are summarized in Table 1. Most of the participants were females (59.2%), nurses (92.5%) and under 30 years old (56.5%). Most of the participants holded only a BSc degree (higher education) (69.9%). More than one third of the participants had 5–10 years of experience (32.5%) and about 15.1% had 1–2 years of experience. Most of them were working in intensive care units (ICU) and ward, 34.6 and 35.3% respectively.

**Table 1:** Participants’ characteristics – questionnaires validation (*N* = 292)

Variable	N (%)
**Age**	
21–30 years old	165 (56.5)
31–40 years old	85 (29.1)
41–50 years old	24 (8.2)
50+ years old	18 (6.2)
**Gender**	
Females	173 (59.2)
**Professional status**	
Nurses	270 (92.5)
Physicians	21 (7.2)
Other	1 (0.3)
**Family status**	
Married	153 (52.4)
Single	129 (44.2)
Divorced	9 (3.1)
Widow/widower	1 (0.3)
**Educational status**	
Higher education	204 (69.9)
Postgraduate diploma (MSc)	85 (29.1)
Doctoral diploma (PhD)	3 (1)
**Total years of clinical experience**	
1–2 years	44 (15.1)
3–5 years	64 (21.9)
5–10 years	95 (32.5)
10+ years	89 (30.5)
**Department of working**	
Intensive care unit (ICU)	101 (34.6)
Ward	103 (35.3)
Emergency department	57 (19.5)
Operation rooms	16 (5.5)
ICU and ward	10 (3.4)
Other	5 (1.7)

### Validity

The CFA of Gr-T-TPQ confirmed the initial scale structure with excellent results regarding the fit on five-factor model (“Team structure”: Items 1–7, “Leadership”: Items 8–14, “Situation monitoring”: Items 15–21, “Mutual support”: Items 22–28 and “Communication”: Items 29–35) (Table 2). Most diagnostic criteria had a very good fit with RMSEA = 0.06 (90% CI, 0.055–0.065), AGFI = 0.986, TLI = 0.994 and CFI = 0.994. The chi-square test for the model was x^2^ (df) = 1124.75 (550), p < 0.0001 (Table 2). In order to indicate a good fit, the chi-square should be not significant although the chi-square statistic is very sensitive to the sample size and is often found to be significant. Kaiser-Meyer-Olkin coefficient for sampling adequacy was 0.652 and Bartlett’s test of sphericity, was high and statistically significant, i.e. 132.611 (*p*-value < 0.001), supporting that the data is appropriate for factor analysis.

**Table 2:** Confirmatory factor analysis of GrT-TPQ

Factors	Team Structure	Leadership	Situation monitoring	Mutual support	Commu-nication
Items					
1. The skills of staff overlap sufficiently so that work can be shared when necessary.	0.61				
2. Staff is held accountable for their actions.	0.61				
3. Staff within my unit share information that enables timely decision-making by the direct patient care team.	0.76				
4. My unit makes efficient use of resources (e.g. staff, supplies, equipment and information).	0.74				
5. Staff understands their roles and responsibilities.	0.84				
6. My unit has clearly articulated goals.	0.77				
7. My unit operates at a high level of efficiency.	0.78				
8. My supervisor/manager considers staff input when making decisions about patient care.		0.81			
9. My supervisor/manager provides opportunities to discuss the unit’s performance after an event.		0.86			
10. My supervisor/manager takes time to meet with staff to develop a plan for patient care.		0.84			
11. My supervisor/manager ensures that adequate resources (e.g. staff, supplies, equipment and information) are available.		0.84			
12. My supervisor/manager resolves conflicts successfully.		0.86			
13. My supervisor/manager models appropriate team behaviour.		0.89			
14. My supervisor/manager ensures that staff is aware of any situations or changes that may affect patient care.		0.86			
15. Staff effectively anticipates each other’s needs.			0.67		
16. Staff monitors each other’s performance.			0.61		
17. Staff exchanges relevant information as it becomes available.			0.78		
18. Staff continuously scans the environment for important information.			0.82		
19. Staff shares information regarding potential complications (e.g. patient changes, bed availability).			0.81		
20. Staff meets to re-evaluate patient care goals when aspects of the situation have changed.			0.74		
21. Staff corrects each other’s mistakes to ensure that procedures are followed properly.			0.80		
22. Staff assists fellow staff during high workload.				0.82	
23. Staff request assistance from fellow staff when they feel overwhelmed.				0.72	
24. Staff cautions each other about potentially dangerous situations.				0.8	
25. Feedback between staff is delivered in a way that promotes positive interactions and future change.				0.82	
26. Staff advocates for patients even when their opinion conflicts with that of a senior member of the unit.				0.43	
27. When staff has a concern about patient safety, they challenge others until they are sure the concern has been heard.				0.65	
28. Staff resolves their conflicts, even when the conflicts have become personal.				0.62	
29. Information regarding patient care is explained to patients and their families in lay terms.					0.61
30. Staff relay relevant information in a timely manner.					0.81
31. When communicating with patients, staff allow enough time for questions.					0.74
32. Staff uses common terminology when communicating with each other.					0.71
33. Staff verbally verifies information that they receive from one another.					0.77
34. Staff follows a standardized method of sharing information when handing off patients.					0.78
35. Staff seeks information from all available sources.					0.79
Goodness-of-fit					
Chi-square (df)	1124.761 (550)				
*p*-value	< 0.0001				
RMSEA	0.06				
90% CI for RMSEA	(0.055–0.065)				
GFI	0.989				
AGFI	0.986				
NFI	0.987				
CFI	0.994				
TLI	0.994				

Factor I, Team structure; Factor II, Leadership; Factor III, Situation monitoring; Factor IV, Mutual support; Factor V, Communication; *RMSEA* root mean square error of approximation; *GFI* goodness-of-fit index; *AGFI* adjusted goodness of-fit index; *NFI* normed fit index; *CFI* comparative fit index; *TLI* Tucker Lewis Index. Levels for an acceptable model fit: RMSEA≤0.08; GFI ≥ 0.90; AGFI≥0.90; NFI ≥ 0.90; CFI ≥ 0.90; TLI ≥ 0.90

The CFA of the Gr-MSQ-short did not confirm the initial scale’s two dimensions (intrinsic and extrinsic). More specifically, RMSEA was found to be equal to 0.12 (90% CI, 0.117–0.133), AGFI = 0.889, CFI = 0.897 and TLI = 0.883. An EFA was performed afterwards with an extraction of two factors. The oblimin rotation with two factors was explaining 58% of the variance (Table 3). Kaiser-Meyer-Olkin coefficient for sampling adequacy was 0.910 and Bartlett’s test of sphericity was found to be significant (X^2^(105) =2583, *p*-value < 0.001), supporting that the data is appropriate for factor analysis. In EFA, items 1, 5, 6, 12 and 18 were eliminated from the analysis due to low communalities and multiple components loading. The two factors identified (Table 3) are: Factor 1, includes the items 2,3,4,7,8,9,10,11 (“The chance to work alone on the job”, “The chance to do different things from time to time”, “The chance to be “somebody” in the community”, “Being able to do things that don’t go against my conscience”, “The way my job provides for steady employment”, “The chance to do things for other people”, “The chance to tell people what to do”, “The chance to do something that makes use of my abilities”) and reflect how empowered and satisfied with supervision HCPs are (Supervisor/Autonomy). Factor 2, includes the items 13,14,15,16,17,19,20 (“My pay and the amount of work I do”, “The chances for advancement of this job”, “The freedom to use my own judgment”, “The chance to try my own methods of doing my job”, “The working conditions”, “The praise I get for doing a good job”, “The feeling of accomplishment I get from the job”) which reflect personal satisfaction HCPs get from their work (Task enrichment). The two-factor model suggested by EFA was supported with a CFA with the following fit indices: RMSEA = 0.08 (90%CI 0.068–0.091), AGFI = 0.906, TLI = 0.916 and CFI = 0.935. The chi-square test for the model was X^2^ (81) =237, p < 0.001 (Table 3). As stated, in order to indicate a good fit, the chi-square should be not significant although the chi-square statistic is very sensitive to the sample size and is very often significant.

**Table 3:** Exploratory factor analysis of GrMSQ-short with an extraction of two factors. Rotated component matrix^2^

	Factors	
Items	Supervisor/ Autonomy	Task Enrichment
2. The chance to work alone on the job	0.73	
3. The chance to do different things from time to time	0.743	
4. The chance to be “somebody” in the community	0.709	
7. Being able to do things that don’t go against my conscience	0.69	
8. The way my job provides for steady employment	0.576	
9. The chance to do things for other people	0.659	
10. The chance to tell people what to do	0.577	
11. The chance to do something that makes use of my abilities	0.722	
13. My pay and the amount of work I do		0.619
14. The chances for advancement of this job		0.799
15. The freedom to use my own judgment		0.83
16. The chance to try my own methods of doing my job		0.728
17. The working conditions		0.805
19. The praise I get for doing a good job		0.93
20. The feeling of accomplishment I get from the job		0.807
**Full Scale Cronbach’s**	0.955	
**Composite Reliability**	0.871	0.921
**Cronbach’s alpha**	0.873	0.888
**Factors correlation**		
Supervisor/ Autonomy	1	
Task Enrichment	0.74	1
Goodness-of-fit		
Chi-Square (df)	237,743(81)	
*p*-value	< 0,0001	
RMSEA	0.08	
90% CI for RMSEA	(0,068 - 0,091)	
TLI	0.916	
NFI	0.905	
CFI	0.935	
GFI	0.906	

*RMSEA* root mean square error of approximation; *GFI* goodness-of-fit index; *AGFI* adjusted goodness-of-fit index; *TLI* Tucker Lewis Index;*NFI* normed fit index; *CFI* comparative fit index

Levels for an acceptable model fit: RMSEA≤0.08,TLI ≥ 0.90;NFI ≥ 0.90,CFI ≥ 0.90

### Reliability

Composite reliability (CR) scores and Cronbach’s alpha coefficients were utilized to provide measures of reliability of the constructs (factors) and internal consistency of individual items, respectively. Hair (2010) recommends a minimum of 0.7 in CR scores [27]. CR scores of the Gr-T-TPQ were found to be high: 0.89 (Team Structure), 0.95 (Leadership), 0.90 (Situation monitoring), 0.87 (Mutual Support) and 0.89 (Communication). The Cronbach’s alpha coefficient of the total scale was also high (0.95). Computed values of Cronbach’s alpha for each of the extracted factors were: 0.857 (Team Structure), 0.93 (Leadership), 0.85 (Situation monitoring), 0.81 (Mutual Support) and 0.85 (Communication). Test-retest reliability was high since bivariate correlations were as follows: 0.863, 0.759, 0.87, 0.674, 0.783 and 0.815 for the Total GrT-TPQ score, Team Structure, Leadership, Situation Monitoring, Mutual Support and Communication, respectively.

CR scores on GrMSQ-short were 0.817 (Supervisor/Autonomy) and 0.921 (Task enrichment) suggesting high reliability of the constructs. A high correlation (0.74) between the two estimated factors was also found. The Cronbach’s alpha coefficient of the total scale was 0.955. Computed values of Cronbach’s alpha for each of the extracted factors were: 0.873 (Supervisor/Autonomy) and 0.888 (Task enrichment). Test-retest reliability was high: 0.926, 0.951 and 0.947 for the Supervisor/ Autonomy, Enrichment and the total GrMSQ-short scale, respectively.

The results of the current study have been presented in 2016 in the Heart Failure Association congress (p.1065) and the abstract has been published in the European Journal of Heart Failure [28].

## Discussion

Job satisfaction has broadly been investigated in health care settings for all HCPs [8–10]. Reduced level of job satisfaction is a major cause of reversals at work, particularly in the area of health care services [9, 10]. On the other hand, teamwork is found to be essential for a safer and more effective health care system while the discourse of teamwork has become a defining feature of healthcare reform [12, 29]. In this study, the psychometric properties of the Gr- T-TPQ and Gr-MSQ-short were tested among a sample of 292 Greek-Cypriot HCPs. The results of the study give the opportunity to researches to use the tools in Greek population and also see how psychometric properties are in different populations and health care systems. This seems to be very important, especially for the T-TPQ which is a quite new tool and has only be validated in USA populations [13, 14].

The results of the CFA model of the GrT-TPQ, showed excellent fit for all the criteria, as proposed by Baker et al. (2010) [17]. This is consistent with the findings of two previous studies, including the study of the developers of the tool, which also confirmed the expected model of the tool [17, 18]. Based on these results, we may conclude that CFA investigations of the Gr-T-TPQ managed to provide evidence of the theoretical dimensionality of the scale, as originally proposed by the developers of the tool.

The results of CFA for the three-factor model of the Gr-MSQ-short, as proposed by Weiss et al. (1967) [19], did not show good fitness criteria. It is consistent to findings from previews studies referring to different kinds of professionals and did not confirm the original model of the tool [20–23].

EFA of the data, suggested two factors, accounting for 58% of the variance. These results contrast with the hypothetical three factor model of Weiss et al. (1967) [19]. Items assigned to the factors of the current study, matched only partially the items fitting in the proposed factors. Specifically, the second factor includes items referred to the freedom to use their own judgment and the chance to try their own methods of doing the job, laying within the first subscale of “intrinsic job satisfaction” of the initial model [19]. The change of that factor to supervisor/autonomy will better represent all items included. Items allocation to the factors are similar to the findings of Martins et al. (2012) [22] in a population of Portuguese hospital workers. The two factors suggested by Martins et al. (2012) included supervisor/ empowerment and task enrichment (Table 4). In that study, a two-factor model was found as well with difference in the allocation of three items, compared to the current study [22].

**Table 4:** Summary of previous factor analysis results

	Original	Martins et al. 2012	Current study
1. Being able to keep busy all the time.	Intrinsic	^a^	^a^
2. You have the opportunity to work alone on work.	Intrinsic	Supervisor/ Empowerment	Supervisor/ Autonomy
3. You have the chance to make different things every day.	Intrinsic	Task enrichment	Supervisor/ Autonomy
4. You have the chance to become someone in the community.	Intrinsic	Supervisor/ Empowerment	Supervisor/ Autonomy
5. The way which my boss manages his staff.	Extrinsic	Supervisor/ Empowerment	^a^
6. The ability of my boss to make decisions.	Extrinsic	Supervisor/ Empowerment	^a^
7. You can do things that are not against your conscience.	Intrinsic	^a^	Supervisor/ Autonomy
8. The way that my work offers stable employment.	Intrinsic	^a^	Supervisor/ Autonomy
9. The opportunity to do things for other people.	Intrinsic	^a^	Supervisor/ Autonomy
10. The chance to tell people what to do.	Intrinsic	Task enrichment	Supervisor/ Autonomy
11. The opportunity to do something that makes me use my capabilities.	Intrinsic	Task enrichment	Supervisor/ Autonomy
12. The department policy applied in practice.	Extrinsic	^a^	^a^
13. My salary and quantity of work I do.	Extrinsic	^a^	Task enrichment
14. Chances of progress in this job.	Extrinsic	^a^	Task enrichment
15. The freedom to use your own judgment.	Intrinsic	Task enrichment	Task enrichment
16. The opportunity of anyone to try their own methods to work.	Intrinsic	Task enrichment	Task enrichment
17. Working conditions.	General	^a^	Task enrichment
18. The way where colleagues work together.	General	^a^	^a^
19. The praise I get when i properly do my job.	Extrinsic	Task enrichment	Task enrichment
20. The sense of accomplishment I get from work.	Intrinsic	^a^	Task enrichment

^a^ Items were eliminated from the analysis due to low communalities or multiple factor loadings

Furthermore, some of the items of the EFA of the current study were eliminated from the analysis due to low communalities and multiple components loading. These were the items 1, 5, 6, 12 and 18 [17–19] from which similar results were found in other studies as well for items 1, 12 and 18 (“Being able to keep busy all the time”, “The way company policies are put into practice”, “The way my co-workers get along with each other”) [20–23]. Recent economic restriction in Cyprus and changes due to the recent introduction of the National HealthCare System, had led to a decrease on the number of HCPs, significantly increasing the same time the workload; both in the private and public sector. In addition to this, the health care system and legislation for nurses in Cyprus, do not allow much professional autonomy and decision making. Finally, communication with co-workers is a competence of the leaders/supervisors of each department or the whole hospital and as a result most HCPs had no interaction and communication with them.

Items 5 and 6 (“The way which my boss manages his staff, “The ability of my boss to make decisions”) were not found to fit well in the current study. This again may be due to the way in which the health care system functions. The supervisor of each department or professional status might not have the competence or adequate education background to lead the department. Usually head nurses get the position due to seniority and not based on subjective criteria that support the competence of leadership. At the same time, decisions and changes in health care settings are made more centrally, by the Ministry of Health and not by the supervisor of the department or the hospital.

The majority of previous studies demonstrated adequate internal consistency of the overall T-TPQ scale among different populations, with a Cronbach’s alpha coefficient ranging from 0.70 to 0.93 [17, 18]. Similar results are found in the current study. Factor score determinacy coefficients indicate that the Gr-T-TPQ has high internal consistently reliability. Further to that, all the items of the scale were deemed important since deletion of any of them reduces the scale’s Cronbach’s alpha.

It is certainly interesting that even though the MSQ-short is a short tool, validation of several versions has revealed different factorial structures and varying internal consistency results [20–23]. The version proposed for the Gr-MSQ-short in this study is also significantly different from the original version as it is five items less and with a different structure. As mentioned before, rather than local health care system differences, these differences may result from the heterogeneity of management approaches and cultural factors. The need of further investigation on the effect of health care systems and cultural differences that may affect HCPs teamwork and job satisfaction is needed [29, 30].

### Limitations/strengths

A limitation of the study was that both scales were tested in a Greek speaking Cypriot population with certain linguistic differences, from the official Greek-spoken and written language. For these reasons, any generalization or interpretation of the results should be done with caution. Moreover, criterion validity has not been assessed for the two tools taking for granted they both evaluate different measures. It is important to mention that the study covered almost all the general public hospitals in the areas controlled by the government of Cyprus and the biggest hospitals on the private sector.

## Conclusions

In general, the Gr-T-TPQ and Gr-MSQ-short are construct-valid instruments for measuring perceptions of teamwork and job satisfaction in Greek speaking HCPs’ population. The psychometric properties yielded explain how the tools may be used in several clinical settings, health professionals and health care systems. They also give the opportunity to researchers to compare results and validation giving more explanations and exploration of possible findings and comparisons.

## Data Availability

The datasets used and/or analyzed during the current study are available from the corresponding author, Ioanna Lakatamitou, Cyprus University of Technology, upon reasonable request.

## Abbreviations

HF: Heart Failure
T-TPQ: TeamSTEPPS Teamwork perceptions questionnaire
MSQ-short: Minnesota Satisfaction Questionnaire “short form”
GrT-TPQ: Greek version of the TeamSTEPPS Teamwork perceptions questionnaire
GrMSQ-short: Greek version of the Minnesota Satisfaction Questionnaire “short form”
HCPs: Health Care Professionals
CFA: Confirmatory Factor Analysis
EFA: Exploratory Factor Analysis
ICU: Intensive care units
CR: Composite reliability

## References

[1] Dyrbye LN, Shanafelt TD, Sinsky CA, Cipriano PF, Bhatt J, Ommaya A, West CP, Meyers D. Burnout among health care professionals: a call to explore and address this underrecognized threat to safe, high-quality care. NAM Perspectives. Washington: Discussion Paper, National Academy of Medicine; 2017. https://nam.edu/burnout-among-health-careprofessionals-a-call-to-explore-and-address-this-underrecognized-threat-to-safe-high-quality-care.

[2] Hall LH, Johnson J, Watt I, Tsipa A, O’Connor DB (2016). Healthcare staff wellbeing, burnout, and patient safety: a systematic review. PLoS One.

[3] Sorenson C, Bolick B, Wright K, Hamilton R (2016). Understanding compassion fatigue in healthcare providers: a review of current literature. J Nurs Scholarsh.

[4] Maslach C, Schaufeli WB, Leiter MP (2001). Job burnout. Annu Rev Psychol.

[5] Alexandrova-Karamanova A, Todorova I, Montgomery A, Panagopoulou E, Costa P, Baban A (2016). Burnout and health behaviors in health professionals from seven European countries. Int Arch Occup Environ Health.

[6] Mijakoski D, Karadzhinska-Bislimovska J, Stoleski S, Minov J, Atanasovska A, Bihorac E (2018). Job demands, burnout, and teamwork in healthcare professionals working in a general hospital that was analysed at two points in time. Open Access Maced J Med Sci.

[7] Mijakoski D, Karadzinska-Bislimovska J, Basarovska V, Montgomery A, Panagopoulou E, Stoleski S (2015). Burnout, engagement, and organizational culture: differences between physicians and nurses. Maced J Med Sci.

[8] Strömgren M, Eriksson A, Bergman D, Dellve L (2016). Social capital among healthcare professionals: a prospective study of its importance for job satisfaction, work engagement and engagement in clinical improvements. Int J Nurs Stud.

[9] Gulavani A, Shinde M (2014). Occupational stress and job satisfaction among nurses. Int J Sci Res.

[10] Azeem SM, Akhtar N (2014). The influence of work life balance and job satisfaction on organizational commitment of healthcare employees. Int J Hum Resour Stud.

[11] Platis C, Reklitis P, Zimeras S (2015). Relation between job satisfaction and job performance in healthcare services. Procedia - Soc Behav Sci.

[12] Gluyas H (2015). Effective communication and teamwork promotes patient safety. Nurs Stand.

[13] Kristensen S, Hammer A, Bartels P, Suñol R, Groene O, Thompson CA (2015). Quality management and perceptions of teamwork and safety climate in European hospitals. Int J Qual Heal Care.

[14] Sheppard F, Williams M, Klein VR. TeamSTEPPS and patient safety in healthcare. J Healthc Risk Manag. 2013:32(3):5–10. 10.1002/jhrm.21099 23335296

[15] Helmreich RL (1984). Cockpit management attitudes. Hum Factors.

[16] Sexton JB, Helmreich RL, Neilands TB, Rowan K, Vella K, Boyden J (2006). The safety attitudes questionnaire: psychometric properties, benchmarking data, and emerging research. BMC Health Serv Res.

[17] Baker DP, Amodeo AM, Krokos KJ, Slonim A, Herrera H. Assessing teamwork attitudes in healthcare: Development of the TeamSTEPPS teamwork attitudes questionnaire. Qual Saf Heal Care. 2010;19(6):e49. 10.1136/qshc.2009.036129 20702444

[18] Keebler JR, Dietz AS, Lazzara EH, Benishek LE, Almeida SA, Toor PA (2014). Validation of a teamwork perceptions measure to increase patient safety. BMJ Qual Saf.

[19] Weiss DJ, Dawis R, England G, Lofquist L. Manual for the Minnesota satisfaction questionnaire. Minnesota Stud Voccational Rehabil. 1967;125. Retrieved from http://vpr.psych.umn.edu/sites/g/files/pua2236/f/monograph_xxii_-_manual_for_the_mn_satisfaction_questionnaire.pdf.

[20] Schriesheim CA, Powers KJ, Scandura TA, Gardiner CC, Lankau MJ (1993). Improving construct measurement in management research: comments and a quantitative approach for assessing the theoretical content adequacy of paper-and-pencil survey-type instruments. J Manage.

[21] Marijani R, Marwa Y (2016). The validation of the Minnesota job satisfaction questionnaire ( MSQ ) in Tanzania : a case of Tanzania public service college. J Asian Afr Stud.

[22] Martins H, Proença MT. Minnesota satisfaction questionnaire: psychometric properties and validation in a population of portuguese hospital workers. Investig e Interv em Recur Humanos. 2014;3:888–97.

[23] Buitendach JH, Rothmann S (2009). The validation of the Minnesota job satisfaction questionnaire in selected organisations in South Africa. SA J Hum Resour Manag.

[24] Raykov T (1997). Estimation of composite reliability for congeneric measures. Appl Psychol Meas.

[25] R Development Core Team (2016). R: A language and environment for statistical computing. R Foundation for Statistical Computing.

[26] Rosseel Y (2012). Lavaan : an R package for structural equation modeling. J Stat Softw.

[27] Hair JF, Anderson RE, Tatham RL, Black WC, Babin BJ, Anderson RE (2010). Multivariate data analysis seventh edition. Pearson Prentice Hall.

[28] Billebeau G, Sadoune M, Polidano E, Merval R, Delcayre C, Kodziszewska KK (2016). Abstracts. Eur J Heart Fail.

[29] Körner M, Wirtz MA, Bengel J, Göritz AS. Relationship of organizational culture, teamwork and job satisfaction in interprofessional teams Organization, structure and delivery of healthcare. BMC Health Serv Res. 2015;15(1). 10.1186/s12913-015-0888-y. 10.1186/s12913-015-0888-y PMC4477418 26099228

[30] Schneider B, Barbera KM, West MA, Topakas A, Dawson JF (2014). Climate and culture for health care performance. The Oxford Handbook of Organizational Climate and Culture.

